# Mood profiles of amateur triathletes: Implications for mental health and performance

**DOI:** 10.3389/fpsyg.2022.925992

**Published:** 2022-11-14

**Authors:** Renée L. Parsons-Smith, Sherry Barkase, Geoff P. Lovell, Veronica Vleck, Peter C. Terry

**Affiliations:** ^1^School of Psychology and Counselling, University of Southern Queensland, Toowoomba, QLD, Australia; ^2^School of Health and Behavioural Sciences, University of the Sunshine Coast, Sippy Downs, QLD, Australia; ^3^Interdisciplinary Centre for the Study of Human Performance, Faculdade de Motricidade Humana, University of Lisbon, Cruz Quebrada-Dafundo, Portugal; ^4^Centre for Health Research, University of Southern Queensland, Toowoomba, QLD, Australia

**Keywords:** triathlete, triathlon, age-group, mood profiles, performance, mental health, BRUMS

## Abstract

Moods have been shown to be predictive of athletic performance and a reflection of mental health status. The aims of our study were (a) to compare pre-race mood scores of triathletes with population norms; (b) to compare pre-race mood scores of triathletes grouped by gender and age bands; (c) to explore whether six distinct mood profile clusters reported in the literature were evident among triathletes and their respective prevalence; (d) to determine if pre-race mood scores predicted triathlon performance; and (e) to interpret our findings in terms of the risk of mental health issues for triathletes. Participants were 592 age-group triathletes (also referred to as recreational or amateur triathletes) who completed the Brunel Mood Scale pre-race and recorded their time goal for the race. Mean mood scores deviated significantly from population norms, with Tension and Vigor scores at the 55th and 54th percentile, respectively, and Depression, Anger, Fatigue, and Confusion scores between the 42nd and 46th percentile. Females reported higher Tension scores than males (*p* < 0.001), and those in the 18–25  years and 26–35 years age bands reported higher Tension scores than those in the 46–55 years age band (*p* < 0.008). Using k-means cluster analysis, six distinct mood profiles were identified, the distribution of which approximated the general population, except for a low prevalence of very negative profiles. Mean scores for Depression and Anger were exceptionally low and only 1.5% of triathletes, compared to the normal prevalence of ~5%, reported an “inverse Everest” profile, which is associated with elevated risk of psychopathology. Mood scores did not predict triathlon performance, assessed by finish time as a percentage of time goal. Results showed an association between triathlon participation and psychological well-being. Findings will inform future investigations of mood responses among triathletes and provide a relevant point of reference for applied practitioners who work with triathletes.

## Introduction

Moods reflect interactions with the world around us that can have a profound effect upon mental health as well as performance in sport and in life (Ruiz and Robazza, 2021). Moods are often conceptualized as a set of transient feelings that provide the emotional backdrop for interactions and are typically seen as being of longer duration and lower intensity than emotions, and not usually attributable to an identifiable cause (Beedie et al., 2005). Moods are often viewed as having a valence dimension that varies from positive (e.g., happy) to negative (e.g., depressed) and an arousal dimension that varies from activation (e.g., vigorous) to deactivation (e.g., fatigued; Russell, 1980).

Mood profiling is a process by which an individual’s scores on a mood scale are plotted against normative scores to create a graphical profile and can be used to identify common patterns of mood responses (Terry, 1995). Several distinct mood profiles have been identified in the sports psychology and sports medicine literature, the most notable of which are known as the iceberg, inverse iceberg, and Everest profiles. The iceberg profile is characterized by an above average Vigor score, combined with below average scores for Tension, Depression, Anger, Fatigue, and Confusion, and has long been associated with positive mental health and good athletic performance (Morgan et al., 1987). The iceberg profile was so named because the shape of the profile resembles an iceberg in that most of the scores sit below the waterline (i.e., the mean score of 50), with only the Vigor score sitting above the mean, just as the largest proportion of an actual iceberg sits below the surface of the water. The inverse iceberg profile is characterized by a below average Vigor score, combined with above average scores for Tension, Depression, Anger, Fatigue, and Confusion, and is associated with overtraining and underperformance among athletes (Budgett, 1998; Urhausen et al., 1998). A third mood profile, termed the Everest profile, is an extremely positive profile characterized by a maximum or almost maximum Vigor score and minimum or almost minimum scores for Tension, Depression, Anger, Fatigue, and Confusion, and is associated with superior performance (Terry, 1995).

Additional profiles have been identified among general and athletic populations that, based on their shape, and continuing the nautical/mountain themes, are referred to as the inverse Everest, shark fin, surface, and submerged profiles (Parsons-Smith et al., 2017; Terry and Parsons-Smith, 2019). The inverse Everest profile is characterized by a low score for Vigor, high scores for Tension and Fatigue, and very high scores for Depression, Anger, and Confusion. The very high scores on these three negative mood dimensions reflect elevated risk of mental health disorders (van Wijk et al., 2013). The shark fin profile is characterized by below average scores for Tension, Depression, Anger, Vigor, and Confusion, combined with a high Fatigue score. This combination of low Vigor and high Fatigue has been shown to be associated with injury risk among athletes (Galambos et al., 2005). The surface profile is characterized by average scores on all mood dimensions and therefore represents a typical profile, although it may be considered sub-optimal for sports requiring high levels of Vigor, including triathlon. The submerged profile is characterized by below average scores on all mood dimensions, which may benefit performance in sports that place a premium on low emotional and physical arousal (e.g., shooting, archery). The four mood profiles described above, together with the iceberg and inverse iceberg profiles have been shown to be evident in different language and cultural contexts, having been replicated among Brazilian, Italian, English, and Chinese-speaking athletes (Parsons-Smith et al., 2017; Quartiroli et al., 2018; Brandão et al., 2021; Terry et al., 2021b) and a representative sample of the Singaporean population (Han et al., 2020). To date, the extent to which each of these six mood profiles are reported by triathletes has not been investigated.

There are several reasons why triathlon, which involves sequential swimming, cycling, and running over a variety of distances (Bentley et al., 2002), is a particularly suitable sport within which to investigate relationships between mood, performance, and mental health. Firstly, the majority of triathletes are active participants over the age of 35 (Rios, 2019), referred to as masters athletes, who are often proposed as a model for successful aging (Lepers, 2020; Borges and Del Vecchio, 2021). In contrast to the ever-increasing levels of sedentarism in the equivalent general population (Owen et al., 2010), and the decreasing number of younger triathletes, the relative number of masters triathletes has grown over time (Lepers et al., 2013). Secondly, the multi-disciplinary training involved in preparing for triathlon competition likely meets the most recent recommended criteria for healthy exercise in masters athletes, being high intensity, endurance- and resistance-based (Borges and Del Vecchio, 2021). Indeed, it has been shown that the decline in endurance performance that normally occurs with age is, to some extent, offset amongst triathletes (Lepers et al., 2013; Piacentini et al., 2019). Thirdly, the swim section of triathlon competition, for example, usually occurs in a lake, river, or the sea, all of which are considered to be nature-based locations (Gladwell et al., 2013) rather than in a swimming pool. Hence, triathlon has the potential to provide mental health benefits beyond those accruing purely from the physical activity involved (Vleck et al., 2014: Vleck, 2018). A recent systematic review and meta-analysis (Brito et al., 2022) reported marginally higher cognitive performance outcomes, lower ratings of perceived exertion, exhaustion, fatigue, and tiredness, and higher levels of vigor, for nature-based activity compared to indoor exercise. Finally, the very fact that triathlon incorporates an age-group system, within which amateur athletes can compete against others of a similar age range (up to World Championship level), means that triathlon provides a unique model within which to research the effect of multidisciplinary exercise on health across the lifespan.

Previous mood profiling research completed among triathletes has unearthed some interesting relationships with health and performance outcomes. For example, Main et al. (2010) found that various combinations of training factors and psychological stressors—monitored weekly via the Perceived Stress Scale (Cohen et al., 1983), Brunel Mood Scale (Terry et al., 1999, 2003), Training Stress Scale (Fry et al., 1994) and Athlete Burnout Questionnaire (Raedeke and Smith, 2001)—predicted symptoms of both illness and injury in age-group triathletes. Further, in a longitudinal prospective training diary-based study, Vleck, 2010 used individual race results of eight British national squad triathletes to retrospectively calculate peak performance norms related to self-assessable symptoms of overtraining (Fry et al., 1994) and profile of mood states-C [POMS-C; a forerunner of the Brunel Mood Scale (BRUMS)] factor scores. The extent to which the weekly values for each distress indicator diverged from the individual athlete’s peak performance norms for those indicators were then modelled together with composite weighted training load scores and self-reports of performance decrement using binary logistic regression. Both the Confusion factor score for 2 and 3 weeks prior, and the Anger factor score for the week before the performance decrement occurred, significantly increased the predictive power of the model. In a similar analysis involving the full group of 63 national squad athletes who participated for varying lengths of time showed that mood scores (when assessed in conjunction with other training status indicators), could predict self-reported overuse injury, 3 days prior to when it occurred, with over 90% accuracy.^1^

Mood profiles have also been shown to be related to triathlon race performance. In mood assessments taken 24 h pre-event, better performing male triathletes (top 50% finish) scored lower on Tension than lesser performing males, and better performing females reported higher Vigor and lower total mood disturbance (TMD) scores than lesser performing females (Bell and Howe, 1988). Similarly, Olympic distance athletes who placed in the top 50% of their age group scored lower on Tension, Depression, Anger, and Fatigue than others in their age group (Vleck and Garbutt, 1996). However, the timing of the mood assessment is important. Mood fluctuations on the Tension and Fatigue subscales were evidenced among Ironman triathletes between baseline, prerace, and postrace time periods (Parry et al., 2011), and mood scores were shown to vary between 2 days prior to, the day before, and race day in Olympic distance competitors (Vleck and Garbutt, 1996).

It has been suggested that the increased potential for in-event mood changes during long-duration sports events, such as triathlon, limits the effectiveness of pre-race mood measures to predict performance (Terry, 1995). The extent to which mood assessed approximately 1 hour pre-race can predict short distance triathlon performance (over the Olympic/Sprint/super sprint distances) among age-group triathletes remains unknown. Terry (1995) also suggested that pre-event mood scores would be more predictive of performance among athletes who were homogeneous in terms of ability and conditioning. In the context of triathlon, this would indicate that the mood-performance relationship would tend to be stronger at the elite, professional level than at the age group, amateur level where there is greater heterogeneity of ability and conditioning. Further, Terry (1995) proposed that the mood-performance relationship would be stronger when the performance measure was self-referenced at an individual level. In triathlon, this can be operationalized by assessing objective performance, in terms of actual finish time, against a meaningful point of reference for individuals, namely a target finish time. This proposition was supported empirically in a meta-analysis of the mood-performance literature (Beedie et al., 2000), which reported larger effects in studies using a self-referenced performance criterion (*M* = 0.37) than in studies using finish time or finish position as a performance measure (*M* = 0.28).

Our study is informed by Morgan’s (1985) mental health model which postulates that a mood profile of high Vigor combined with low Anger, Confusion, Depression, Fatigue, and Tension (an iceberg or Everest profile), is indicative of positive mental health, whereas a mood profile of low Vigor combined with high Anger, Confusion, Depression, Fatigue, and Tension (an inverse iceberg or inverse Everest profile), is associated with poor mental health outcomes.

To sum up, triathlon is an extremely demanding sport, both physically and mentally. Although triathlon appears to be a relatively safe activity once high-risk individuals are screened out (Vleck, 2009; Vleck and Hoeden, 2020), and its athletes have been proposed as a potential model for successful aging (Lepers, 2020), health issues such as overuse injury, illness, fatigue, and burnout may also be highly prevalent in this population. Such issues have the potential to overshadow the numerous physical and mental health benefits that are associated with the sport (Vleck et al., 2014). It is therefore seen as a priority that triathlon should have a greater focus on healthy participation, to effectively “futureproof” the sport (Kennedy et al., 2020). With this imperative in mind, and unlike previous research, our study investigated triathletes’ pre-race mood responses from both a performance and mental health perspective. More specifically, we investigated mood responses among a sample of age-group triathletes to address the following research aims: (a) to compare pre-race mood scores of triathletes with population norms; (b) to compare pre-race mood scores of triathletes grouped by gender and age bands; (c) to explore whether six distinct mood profile clusters reported in the literature were evident among triathletes and their respective prevalence; (d) to determine if pre-race mood scores predicted triathlon performance; and (e) to interpret our findings in terms of the risk of mental health issues for triathletes.

## Materials and methods

### Participants

A total of 592 age-group triathletes participated in the study and provided complete data (male identity = 377; female identity = 200; other/not specified = 15; age range 18–81 years, *M*_age_ = 39.86 years, *SD*_age_ = 11.11 years). The study participants were grouped into five age bands (18–25 years, *n* = 48; 26–35 years, *n* = 164; 36–45 years, *n* = 207; 46–55 years, *n* = 122; 56+ years, *n* = 51) to facilitate a comparison of mood responses by age. These age bands did not correspond to those used by race organizers, which are typically split into 5-year bands (20–24 years, 25–29 years, 30–34 years, etc.), but were instead chosen so that each age band had sufficient participants to maximize the probability that all six hypothesized mood profiles would be evident. Of those participants who indicated their ethnicity, 94.7% (523 of 552) identified themselves as Caucasian. Of those who were approached to participate, the acceptance rate was approximately 90%.

### Measures

#### Mood assessment

Mood was assessed using the BRUMS (Terry et al., 1999, 2003). Originally adapted from the Profile of mood states (POMS; McNair et al., 1971), the BRUMS has six subscales of four items each (i.e., Tension—items nervous, anxious, worried, panicky; Depression—items unhappy, miserable, depressed, downhearted; Anger—items bitter, angry, annoyed, bad tempered; Vigor—items energetic, active, lively, alert; Fatigue—items exhausted, tired, worn out, sleepy; and Confusion—items mixed up, muddled, uncertain, confused). Participants rated each mood descriptor on a 5-point Likert-type scale of 0 = not at all, 1 = a little, 2 = moderately, 3 = quite a bit, and 4 = extremely. Subscale scores range from 0 to 16. The response timeframe used was “How do you feel right now?” to capture pre-race mood. In the original validation studies, the BRUMS demonstrated robust psychometric properties using multi-sample confirmatory factor analysis that supported the configural, metric, scalar, and residual invariance of the measurement model across samples of adult students, adult athletes, young athletes, and schoolchildren (Terry et al., 1999, 2003). The BRUMS has demonstrated satisfactory internal consistency, with Cronbach alpha coefficients (Cronbach, 1951) ranging from 0.74–0.90 for the six subscales (Terry et al., 2003). The BRUMS does not provide a comprehensive assessment of the global domain of mood (Ekkekakis, 2013), so researchers using the measure are cautioned not to extrapolate findings beyond the six specific mood dimensions assessed. The BRUMS has been used in many mental health settings, for example, to assess adolescents for elevated suicide risk in the USA (Gould et al., 2005); to screen for risk of post-traumatic stress disorder among military personnel in South Africa (van Wijk et al., 2013); to manage performance anxiety and prevent injuries among adolescent ballet dancers in Japan (Yatabe et al., 2014); and to evaluate population-level mental health and monitor the psychological well-being of cardiac rehabilitation patients in Brazil (Sties et al., 2014; Brandt et al., 2016).

#### Performance measure

Participants provided their race number, which allowed their finish time to be obtained from the official race websites. Participants also provided their time goal for the event. Based on the empirical evidence provided by Beedie et al. (2000), which demonstrated the benefit of self-referenced performance indicators, the performance measure used for analysis was objective, self-referenced, and individualized, calculated using finish time as a percentage of time goal. Objective, self-referenced, and individualized performance measures provide a more sensitive indicator of the quality of performance than absolute measures of finish time because they account for individual differences in ability and conditioning (Terry, 1995). Performance scores above 100% represented a finish time for an individual that was slower than their time goal. Performance scores at or below 100% represented a finish time that equaled or bettered their time goal.

### Procedure

The design of this investigation was a quantitative, non-experimental, cross-sectional analysis of self-reported and objective variables. Following institutional ethics approval (Human Research Ethics Committee; approval number H18REA170) triathletes over 18 years of age participating at one of three major triathlons held on the Sunshine Coast in Queensland, Australia, were invited to take part in this research as volunteers. All potential participants were briefed regarding the background and relevance of the research. Participants who agreed to take part in this research confirmed that they understood their rights and obligations as a participant and consented to participate in completion of a paper-based survey. This study was carried out in accordance with the recommendations of the Australian Code for the Responsible Conduct of Research.

Data were collected at the 2018 Noosa Triathlon and Multisport Festival as well as the 2019 and 2020 Mooloolaba Triathlons. These are high-profile triathlons that include both elite races and large-scale (4,000+ competitors) age-group events over the Olympic distance (1.5 km swim/40 km cycle/10 km run) or Sprint distance (750 m swim/20 km bike/5 km run). All three events offered both individual and team relay (male, female, or mixed) races over the Olympic distance. In the Mooloolaba 2020 event, the swim and swim-bike transition distances were shortened due to poor weather conditions, of which all athletes were advised in advance. Participants were approached by members of the data collection team (27 volunteer members) whilst waiting for their event to start. Data were collected, on average, within 1 h. of the race commencing (*M* = 55 min, SD = 38 min.).

### Data analyses

Analyses were conducted using SPSS Statistics for Windows, Version 28 (IBM IBM Corporation, 2021). A power analysis using G*Power 3 (Faul et al., 2007) indicated that the minimum sample size required to detect even small effects with statistical power of at least .8 and an alpha level of .05 was 327 participants. Our sample of 592 participants exceeded this recommended minimum, indicating that our analyses had adequate statistical power. All cases with missing data were excluded from the analyses and complete data were checked for non-normality. Positive skewness was apparent for Anger and Depression scores, indicating a high proportion of low scores and a long tail towards the upper end of these scales, which is typically found for measures of negative moods (Terry et al., 1999, 2003). Kurtosis values were high for Anger, Confusion, and Depression scores, which again is commonly found for these subscales (Terry et al., 1999, 2003). Usually, the full range of scores from 0 to 16 for each BRUMS subscale is recorded within study samples (Terry et al., 2003). However, among triathletes in the present sample, this was only the case for the Tension and Vigor subscales. No scores above 12 were reported for Fatigue and Confusion, only one score above 12 was reported for Anger, and only three scores above seven were reported for Depression. Indeed, 85% of triathletes reported a zero score for Depression and 81% reported a zero score for Anger, being exceptionally low figures. A total of 11 significant multivariate outliers were identified using the Mahalanobis statistic (*p* < 0.001), although a case-by-case inspection found no examples of response bias in the form of acquiescent, extreme, or straight-line responding (Meisenberg and Williams, 2008; Leiner, 2019) and therefore no data transformations occurred. Cronbach alpha coefficients in the present study ranged from 0.78–0.85 except for the Confusion subscale at 0.67, marginally lower than the prescribed level of acceptability (Nunnally, 1978).

**Figure 1:**
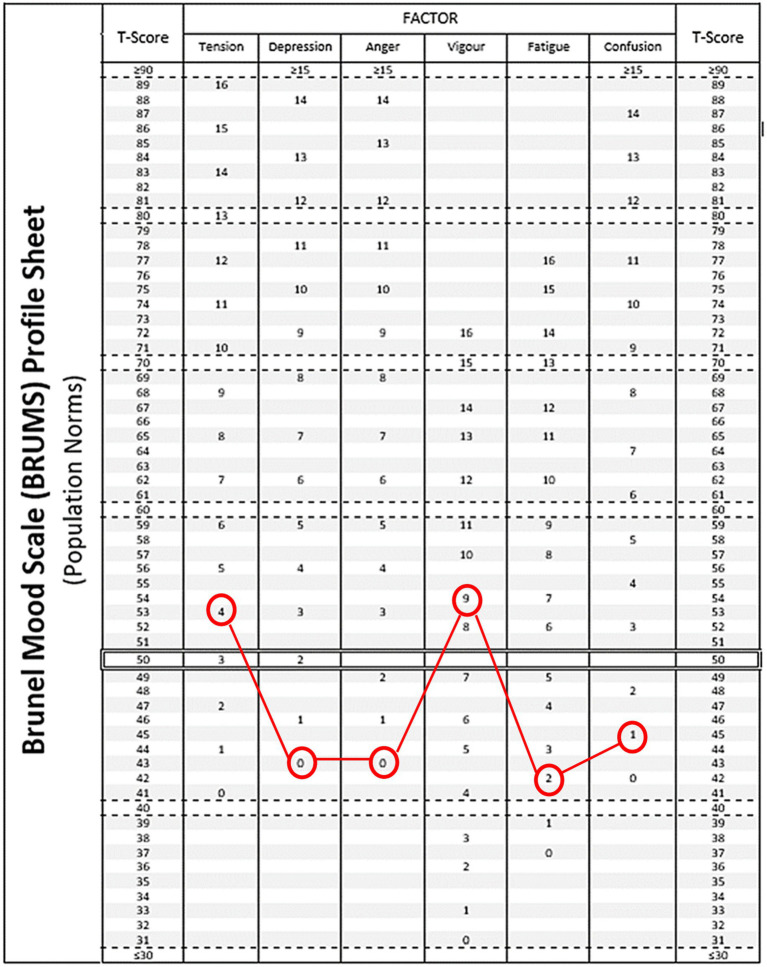
Mean mood profile for age-group triathletes (*N* = 592) plotted against population norms from Terry and Parsons-Smith (2021).

To address the first research aim, raw scores for the BRUMS were converted into standard scores (T-scores) using the table of population normative data (*N* = 15,692; Terry and Parsons-Smith, 2021). The observed mean T-scores for the six mood dimensions of the BRUMS were then plotted against those population norms to check for similarity (Figure 1). To address the second research aim, two single-factor MANOVAs with *post hoc* pairwise comparisons were conducted to establish whether mood scores varied by gender and age band. Partial *η^2^* effect sizes were calculated for each significant pairwise comparison to assess explained variance (Barry et al., 2016). To address the third research aim, which was to establish whether the six mood profile clusters reported in the literature (Parsons-Smith et al., 2017) were also evident among triathletes, and their respective prevalence, a seeded k-means cluster analysis with a prescribed 6-cluster solution was conducted. A *post hoc* simultaneous multiple discriminant function analysis based on unequal group sizes was used to evaluate how accurately cluster membership was predicted. Chi-square tests were used to detect whether the mood profile distributions differed by gender and age band. To address the fourth research aim, the predictive effectiveness of pre-race mood on performance was explored using bivariate correlations between performance scores and mood scores, followed by linear regression analysis to predict performance variance from the six mood scores collectively.

## Results

### Comparison with normative scores

The mean triathlete mood profile deviated significantly from the population norms, and one-sample t-tests confirmed significant differences between the observed mean scores and a normative mean of 50 (SD = 10; see Table 1). The magnitude of the differences ranged from moderate for higher Tension and Vigor and lower Confusion, to very large for lower Depression, Anger, and Fatigue (Terry and Parsons-Smith, 2021). Further, and again in comparison with the population normative data, a much higher number of triathlon participants scored zero for Depression (85.0% vs. 47.9%), Anger (80.9% vs. 44.5%), Fatigue (34.8% vs. 11.0%), and Confusion (43.9% vs. 37.8%).

**Table 1:** Mean BRUMS scores for triathletes (*N* = 592) vs. norms (*N* = 15,692).

Subscale	*M*	SD	95% CI	*t*	*d*
Tension	54.74	10.33	[53.90, 55.57]	11.16^†^	0.46
Depression	44.28	4.33	[43.93, 44.63]	−32.13^†^	1.32
Anger	44.41	4.37	[44.05, 44.76]	−31.14^†^	1.28
Vigor	53.70	8.20	[53.04, 54.36]	10.98^†^	0.45
Fatigue	42.31	5.90	[41.83, 42.79]	−31.71^†^	1.30
Confusion	46.66	6.24	[46.16, 47.16]	−13.03^†^	0.54

All scores are T-scores; *t*, *t*-test for difference between observed mean and normative mean of 50 (SD = 10); *d*, effect size.

† *p* < 0.001.

### Mood responses by gender and age

Multivariate main effects were found for both analyses (gender: partial *η^2^* = 0.057; age band: partial *η^2^* = 0.022). Follow-up univariate and pairwise comparisons identified significant between-group differences on the Tension subscale (partial *η^2^* = 0.044). Having applied a Bonferroni adjustment to the alpha level, Tension scores were higher among females than males (*p* < 0.001), and higher among the 18–25 years and 25–35 years groups than the 46–55 years group (*p* < 0.008). Participant characteristics, descriptive statistics, and between-group comparisons are shown in Table 2.

**Table 2:** Participant characteristics and between-group comparisons.

Source	*n* (%)	*M*	SD	95% CI
Gender (Wilks’*Λ* = 0.931, *F*_(6, 570)_ = 7.04^†^, partial *η^2^* = 0.069)				
Male^a^	377 (65.3)			
Tension		48.35^†^^b^	8.89	[47.36, 49.33]
Depression		50.25	10.64	[49.22, 51.27]
Anger		50.13	10.17	[49.11 51.16]
Vigor		50.08	10.21	[49.08, 51.09]
Fatigue		50.20	10.09	[49.19, 51.22]
Confusion		49.59	9.18	[48.58, 50.61]
Female^b^	200 (34.7)			
Tension		53.36^†^^a^	11.17	[52.01, 54.71]
Depression		49.71	9.12	[48.30, 51.12]
Anger		49.88	10.05	[48.48, 51.29]
Vigor		49.76	9.40	[48.38, 51.15]
Fatigue		49.81	9.99	[48.41, 51.21]
Confusion		51.00	11.51	[49.61, 52.40]
Total	577 (100)			
Age band (year**)** (Wilks’Λ = 0.913, *F*_(24, 2032)_ = 2.23^†^, partial *η^2^* = 0.022)				
18–25^c^	59 (10.0)			
Tension		53.88^§^^f^	9.45	[51.38, 56.39]
Depression		51.73	14.84	[49.18, 54.29]
Anger		51.45	11.35	[48.89, 54.01]
Vigor		52.63	11.10	[50.10, 55.16]
Fatigue		50.08	8.69	[47.53, 52.62]
Confusion		52.56	11.48	[50.01, 55.10]
26–35^d^	153 (25.8)			
Tension		51.97^§^^f^	10.28	[50.41, 53.52]
Depression		48.43	5.22	[46.85, 50.02]
Anger		49.22	7.70	[47.63, 50.81]
Vigor		49.42	8.80	[47.85, 51.00]
Fatigue		50.87	10.25	[49.29, 52.45]
Confusion		50.98	9.96	[49.39, 52.56]
36–45^e^	207 (35.0)			
Tension		49.54	10.62	[48.20, 50.88]
Depression		50.20	9.94	[48.83, 51.56]
Anger		50.52	10.80	[49.15, 51.89]
Vigor		49.94	9.51	[48.60, 51.29]
Fatigue		50.93	10.90	[49.57, 52.29]
Confusion		49.96	10.46	[48.60, 51.32]
46–55^f^	122 (20.6)			
Tension		47.47^§^^cd^	8.07	[45.72, 49.21]
Depression		50.67	12.09	[48.89, 52.45]
Anger		49.55	10.91	[47.77, 51.34]
Vigor		49.77	10.07	[48.02, 51.53]
Fatigue		48.74	9.34	[46.97, 50.51]
Confusion		48.58	9.15	[46.81, 50.35]
56+^g^	51 (8.6)			
Tension		47.58	8.99	[44.89, 50.28]
Depression		50.42	8.58	[47.67, 53.18]
Anger		49.75	9.05	[46.99, 52.51]
Vigor		49.55	12.42	[46.83, 52.27]
Fatigue		46.75	7.49	[44.01, 49.49]
Confusion		47.87	7.55	[45.13, 50.61]
Total	592 (100)			

Superscript letters indicate significant between-group differences.

§ *p* < 0.008;

† *p* < 0.001.

### Cluster analysis

**Figure 2:**
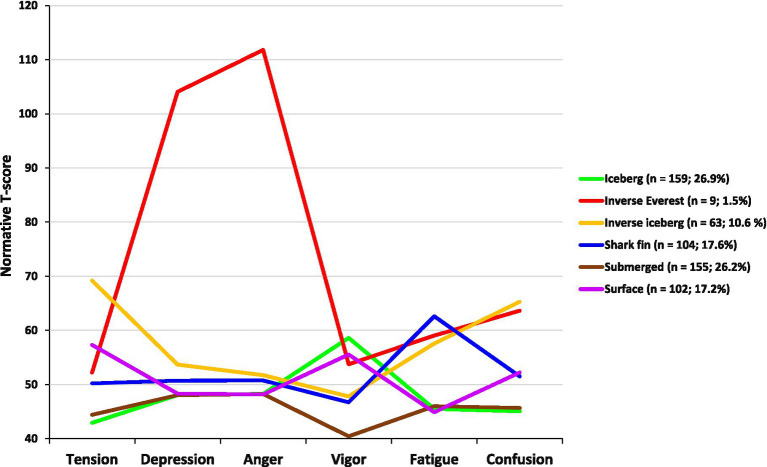
Graphical representation and prevalence of the six mood clusters.

The six clusters reported in the literature were confirmed in our sample. In descending order, the prevalence of the six mood profiles among our sample of triathletes was iceberg (26.9%), submerged (26.2%), shark fin (17.6%), surface (17.2%), inverse iceberg (10.6%), and inverse Everest (1.5%; Figure 2). Descriptive statistics for each of the mood profiles are shown in Table 3. A total of 93.9% of cases were reclassified into their original groupings, consistent with the high classification accuracy rates found in previous samples (Parsons-Smith et al., 2017; Quartiroli et al., 2018; Terry and Parsons-Smith, 2019). The classification accuracy rates for each cluster were: iceberg profile = 93.7%, inverse Everest profile = 100%, inverse iceberg profile = 93.7%, shark fin profile = 88.5%, submerged profile = 96.1%, and surface profile = 96.1% (see Table 4).

**Table 3:** Descriptive statistics of the six-cluster solution.

	Iceberg (*n* = 159; 26.9%)			Inverse Everest (*n* = 9; 1.5%)			Inverse iceberg (*n* = 63; 10.6%)			Source
	*M*	SD	95% CI	*M*	SD	95% CI	*M*	SD	95% CI
Tension	42.92	4.33	[42.24, 43.60]	52.19	9.84	[44.63, 59.76]	69.19	6.44	[67.57, 70.81]
Depression	48.05	5.16	[47.25, 48.86]	104.05	32.82	[78.82, 129.27]	53.68	11.18	[50.86, 56.49]
Anger	48.25	5.65	[47.37, 49.14]	111.80	19.52	[96.80, 126.80]	51.73	8.30	[49.64, 53.82]
Vigor	58.61	6.21	[57.63, 59.58]	53.75	12.89	[43.84, 63.66]	47.80	9.61	[45.38, 50.22]
Fatigue	45.49	5.56	[44.62, 46.36]	59.04	18.46	[44.85, 73.22]	57.57	11.09	[54.77, 60.36]
Confusion	45.06	4.79	[44.31, 45.81]	63.64	12.78	[53.82, 73.46]	65.28	14.13	[61.73, 68.84]
	Shark fin (*n* = 104; 17.6%)			Submerged (*n* = 155; 26.2%)			Surface (*n* = 102; 17.2%)			Source
	*M*	SD	95% CI	*M*	SD	95% CI	*M*	SD	95% CI
Tension	50.19	6.22	[48.98, 51.40]	44.41	5.56	[43.53, 45.29]	57.32	4.93	[56.35, 58.28]
Depression	50.72	7.68	[49.22, 52.21]	48.03	3.85	[47.42, 48.64]	48.31	4.06	[47.51, 49.11]
Anger	50.75	6.98	[49.39, 52.10]	48.25	5.19	[47.42, 49.07]	48.17	4.25	[47.33 49.00]
Vigor	46.72	6.46	[45.47, 47.98]	40.44	6.05	[39.48, 41.40]	55.54	6.54	[54.26, 56.83]
Fatigue	62.61	8.49	[60.96, 64.26]	45.99	5.59	[45.12, 46.88]	44.89	4.97	[43.92, 45.87]
Confusion	51.49	8.51	[49.84, 53.14]	45.66	4.60	[44.93, 46.39]	52.23	8.50	[50.56, 53.89]

**Table 4:** Cluster classifications (*N* = 592).

	Predicted group membership						
Cluster	1	2	3	4	5	6	*n*
Iceberg	149	3	0	4	2	1	159
Inverse Everest	0	9	0	0	0	0	9
Inverse iceberg	0	0	59	3	0	1	63
Shark fin	2	2	1	92	6	1	104
Submerged	0	3	1	1	149	1	155
Surface	1	1	0	2	0	98	102

1 = Iceberg profile, 2 = Inverse Everest profile, 3 = Inverse iceberg profile, 4 = Shark fin profile, 5 = Submerged profile, 6 = Surface profile.

### Prevalence of mood profile clusters by gender and age

Results showed that females were underrepresented in the iceberg profile while males were underrepresented in the inverse iceberg and surface profiles (see Table 5). For age band, triathletes aged 46–55 years were overrepresented for the iceberg profile, whereas those aged 25–35 years were underrepresented. The 25–35 years group was overrepresented for the inverse iceberg profile, whereas the 46–55 years group was underrepresented. For the shark fin profile the 36–45 years group was overrepresented. For the submerged profile, the 46–55 years group was overrepresented, whereas the 25–35 years group was overrepresented. No gender and age differences were evident for the surface profile.

**Table 5:** Distribution of six mood profile clusters by gender and age band.

	Cluster					
Source	1	2	3	4	5	6
Gender [*χ*^2^_(5)_ = 25.57^†^]						
Male	113	6	29^§^–	71	106	52^§^–
Female	40^§^–	3	33	30	45	49
Total (*n* = 577)	153	9	62	101	151	101
Age band (year) [*χ*^2^_(20)_ = 43.79^§^]						
18–24	15	2	7	7	4^§^–	13
25–35	32^*^–	1	27^§^^+^	30	38	36
36–45	54	3	20	45^*^^+^	55	30
46–55	42^*^^+^	2	7^*^–	15	42^*^^+^	14
56+	16	1	2	7	16	9
Total (*N* = 592)	159	9	63	104	155	102

1 = Iceberg profile, 2 = Inverse Everest profile, 3 = Inverse iceberg profile, 4 = Shark fin profile, 5 = Submerged profile, 6 = Surface profile; +over-represented, −under-represented.

* *p* < 0.05;

§ *p* < 0.01;

† *p* < 0.001.

### Pre-race mood and triathlon performance

The non-elite status of our sample was confirmed by their modest finish times compared to elite triathletes. The fastest elite times for the Olympic distance events were < 1 h. 50 min. for men and just over 2 h. for women. Among our sample, the mean finish time for individuals completing Olympic distance events (Noosa 2018 and Mooloolaba 2019) was 2 h. 55 min. 26 s (SD = 25 min. 55 s, *n* = 321), whereas the mean finish time for individuals completing the shortened Olympic distance event (Mooloolaba 2020) was 2 h. 40 min. 13 s (SD = 22 min. 10 s, *n* = 179). The mean finish time for individuals completing the Sprint event was 1 h. 36 min. 25 s (SD = 12 min. 13 s, *n* = 44). Among team competitors, the mean finish times were 23 min. 29 s for the swim leg (*SD* = 8 min. 34 s, *n* = 10), 1 h. 21 min. 26 s for the cycle leg (*SD* = 12 min. 20 s, *n* = 15), and 59 min. 0 s for the run leg (SD = 9 min. 18 s, *n* = 16). Seven team competitors did not specify which leg they were completing. The wide range of finish times among our sample highlights the importance of using a performance metric that references actual finish time against the individual triathlete’s target finish time.

A total of 41.7% of individual competitors and 47.9% of team competitors equaled or bettered their time goal, meaning that most participants did not achieve their target finish time. Bivariate correlations showed no significant relationships between the performance measure (i.e., finish time as a percentage of time goal) and any mood factor score (*r* range: −0.06 to .08, *p* > 0.05) and linear regression analysis showed that, collectively, mood scores predicted only 1% of variance in triathlon performance (*F*_6,585_ = 1.95, adjusted *R*^2^ = 0.01, *p* > 0.05). Overall, our results showed no significant predictive effectiveness of pre-race mood scores on performance in triathlon, even though the performance measure was objective, self-referenced, and individualized.

## Discussion

In response to the limited research that has investigated the moods of triathletes from the combined perspective of performance and mental health, our study utilized participants’ pre-event mood and predicted performance time goal to address four research aims. In answer to the first research aim, it was found that the mean pre-competition mood profile of the triathletes differed significantly from general population normative scores. Our triathlete sample had meaningfully (moderate to large effect sizes) higher Tension and Vigor with lower Depression, Anger, Fatigue, and Confusion scores than typically found in the general population (Terry and Parsons-Smith, 2021). Despite the elevated Tension scores in this sample, we interpreted the observed mood profiles as representative of a clear association between positive mood and triathlon participation. This contention was further supported by the much higher than normal number of triathlon participants who scored zero for Depression, Anger, Fatigue, and Confusion, compared to the general population (Terry and Parsons-Smith, 2021). These differences in mood scores between our sample of triathletes and normative scores for the population were of sufficient magnitude to suggest that separate triathlon-specific tables of normative data for the BRUMS should be generated as a future research direction.

An explanation for the higher Tension scores in this sample of triathletes is the likely effect of data being collected in a pre-competition environment. Previous research with Ironman triathletes has shown significant increases in Tension scores associated with competition (Parry et al., 2011) and, among Olympic distance athletes, Vleck and Garbutt, (1996) reported significantly higher Tension scores on race day for 55 competitors in the 1995 International Triathlon Union World championships compared to 1 and 2 days prior. Although our participants were recreational rather than elite triathletes, it is likely that pre-competition anxiety and a preoccupation with thoughts associated with competing and/or completing the event, contributed to increased feelings of tension. Our results are also consistent with the findings of Burgum and Smith (2021) who reported a significant increase in Tension scores immediately prior to racing among ultradistance runners.

To address our second research aim, further examination of Tension data showed gender and age differences. Those identifying as females reported higher Tension scores than those identifying as males, and participants in the 18–25 years and 25–35 years age bands reported higher Tension scores than those in the 46–55 years age band. These results are broadly in agreement with the literature. The mood literature often shows gender-based differences, with females typically reporting higher scores for Anger, Confusion, Depression, Fatigue, and Tension than males and lower scores for Vigor (Terry et al., 2020, 2021a). In the present study, gender-based differences in mood scores were more limited, accounting for just 6.9% of variance, with only the difference in Tension scores reaching statistical significance. In the triathlon-specific literature, Dolan et al. (2011) reported that among 401 triathletes, significantly more of whom were females than males, the most common emotion experienced in the hour pre-race was anxiety/nervousness. Hammermeister and Burton (1995) found among a sample of 318 endurance athletes, 185 of whom were triathletes, that males and females reported different perceptions of perceived threat, which may manifest as differences in Tension scores.

As for age-based differences, the mood literature shows a general trend of less negative moods among older adults compared to younger adults (Terry et al., 2021b; Ferrari et al., 2022), which have typically been explained in terms of better developed coping and mood regulation strategies among older adults. For example, younger adults tend to engage more in maladaptive coping strategies, such as rumination, avoidance, and suppression, than older adults (Aldao et al., 2010). Similarly, mindfulness facilitates effective emotion regulation and psychological well-being (Bravo et al., 2016; Remmers et al., 2016) and older adults are more likely to be classified into high mindfulness groups than their younger counterparts (Ford et al., 2020). However, our findings indicated that only 2.2% of the variance in mood scores was associated with age, and only Tension scores differed significantly by age band. The higher Tension scores reported by younger participants might also be explained by them having fewer and less well-developed triathlon-relevant coping and pacing strategies compared to the older participants. Another explanation might relate to the lower ego investment and competition orientation reported by older athletes compared to younger athletes (Molanorouzi et al., 2015).

Regarding our third research aim, all six mood profile clusters reported previously (Parsons-Smith et al., 2017; Quartiroli et al., 2018; Terry et al., 2021b) were evident in the current triathlon sample. Profile prevalence, in descending order, was the iceberg (26.9%), submerged (26.2%), shark fin (17.6%), surface (17.2%), inverse iceberg (10.6%), and inverse Everest (1.5%) profiles. Consistent with previous research, classification accuracy rates were high, ranging between 88.5 and 100%. Although gender and age differences in profiles were observed, the most striking feature of our findings was the extent to which the prevalence rates of the profile clusters differed in this sample compared to other published datasets. In particular, the most negative profile, the inverse Everest profile (characterized by low Vigor, high Tension and Fatigue, and very high Depression, Anger, and Confusion scores), had an extremely low prevalence (1.5%) compared to the general population (4.6%; Terry et al., 2021b) and to athletic samples from Brazil (4%; Brandão et al., 2021), China (7%; Terry et al., 2021c), Italy (5%; Quartiroli et al., 2018), and Singapore (4%; Han et al., 2020).

Viewed in conjunction with the prevalence of the next most negative mood profile, the inverse iceberg profile (characterized by a below average Vigor score, and above average scores for Tension, Depression, Anger, Fatigue, and Confusion), the combined prevalence of the two most negative profiles (i.e., inverse Everest and inverse iceberg) was 12.1% in our triathlete sample, which compared favorably to athlete samples in China (23.1%; Terry et al., 2021c), Italy (19.0%; Quartiroli et al., 2018), Singapore (14.0%; Han et al., 2020), and on a par with athletes in Brazil (12.3%; Brandão et al., 2021). It should be noted that the inverse iceberg and inverse Everest mood profiles are risk factors for a variety of undesirable health outcomes among athletes, including overtraining syndrome (Budgett, 1998), and athletic injury (Galambos et al., 2005). In other domains, the inverse iceberg and inverse Everest mood profiles are risk factors for post-traumatic stress disorder (van Wijk et al., 2013), and, given that both profiles feature high scores for Depression, Anger, and Confusion, they are also associated with increased risk of clinical mood disorders (Ferrari et al., 2022).

To maximize performance, elite and recreational triathletes alike must engage in regular rigorous multidisciplinary training sessions that can predispose them to negative psychological health (e.g., mood disturbance, burnout) and physical health (e.g., injury) consequences (Main et al., 2010; Vleck and Garbutt, 1998; Vleck et al., 2014). Despite this acknowledged risk, the low prevalence of the most negative mood profiles suggests a clear indication that triathlon participation has an association with positive mental health.

Findings related to our fourth research aim demonstrated that in this sample of triathletes, there was no significant relationship between pre-race mood and objective, self-referenced, individualized performance outcome, with only 1% of performance variance being predicted from mood scores. Although research has previously demonstrated a relationship between mood and subsequent athletic performance (Beedie et al., 2000; Lochbaum et al., 2021), the lack of an observed relationship between pre-event mood and performance in triathlon is consistent with the prediction of Terry (1995) that the mood-performance relationship is moderated by event duration. Sports of longer duration are more susceptible to in-event mood fluctuations compared to short duration sports thereby reducing the predictive effectiveness of pre-event mood on subsequent performance. Furthermore, in triathlon, there are a multitude of factors that can vary between and during the three performance elements of the sport (swim, cycle, run), all with the potential to affect performance (Olcina et al., 2018). Examples include race dynamics and changing environmental conditions (temperature, humidity, precipitation, wind velocity and direction, topography; Wu et al., 2014); mechanical reliability issues; nutritional strategy efficacy; unexpected gastrointestinal challenges; injury; and even crashes. These highly variable factors, each with strong potential to substantially disrupt performance, have great capacity to overshadow the effects of pre-event mood upon performance outcomes, perhaps explaining why mood did not associate with performance in this triathlete sample. A recent study of mood fluctuations *during* ultrarunning (Burgum and Smith, 2021), which reported significant in-event increases in Anger, Fatigue and TMD scores, both confirmed the potential for in-event mood fluctuations to occur and supported the benefit of mood stability on performance among endurance athletes.

Turning to our final research aim, interpreting our findings in terms of the mental health of triathletes, a key conclusion based on the low prevalence of the most negative mood profiles, compared to population norms and other sport samples, is that participation in triathlon is associated with decreased risk of mental ill health; this finding is very encouraging for those who participate in the sport of triathlon (Vleck et al., 2014). Explanation of why triathlon participation is associated with protective mental health benefits is complex. Theoretical explanations of the reported positive psychological outcomes of exercise and physical activity are somewhat limited (Daley, 2008). Common physiological explanations include improvements in blood flow efficiency; enhanced “feel good” neurotransmitters (e.g., norepinephrine, endorphins, serotonin); increased maximal oxygen consumption and associated delivery of oxygen to cerebral tissues; reduction in muscle tension; and positive structural changes in the brain (Vleck et al., 2014). Psychological explanations of the positive well-being outcomes associated with exercise include enhanced feelings of control; exercise as a “time-out” or distraction from distressing tasks (Gleser and Mendelberg, 1990); improved self-concept and self-esteem (Taylor and Fox, 2005); and opportunities for fun and enjoyment (Biddle and Mutrie, 2008). Further explanation for the mental health benefits of the sport may also be offered by the Basic Needs Theory (BNT; Deci and Ryan, 2000; Huntsman et al., 2018) in that triathlon meets the three basic self-deterministic needs of autonomy (by virtue of being a primarily individual sport that tests a person to their limit), competence (by allowing even marginal gains to be tracked objectively), and relatedness (by providing a global community of like-minded individuals). Whatever the mechanisms for the positive psychological outcomes associated with physical activity, the results of the current investigation provide evidence that psychological well-being benefits are likely to be derived from triathlon participation.

We acknowledge that our research has limitations. The design of this investigation was cross-sectional and as such prevents assessment of cause and effect. Although this design did facilitate a large sample size, future investigation could adopt longitudinal repeated-measures designs. A further potential limitation was the use of a self-report method to assess mood, although such methods are used with increasing frequency (Everhart et al., 2020) and are considered superior to objective measures in some sport contexts (Saw et al., 2016). Nevertheless, it is acknowledged that all self-report methods are vulnerable to social disability bias. Additionally, it is possible that there was a selection bias effect, whereby participants with more positive mood may have been more likely to complete the survey (Rosenman et al., 2011).

There are several potential directions for future research in this area. Firstly, the magnitude of the differences in mood scores between our sample of triathletes and normative scores for the general population (Terry and Parsons-Smith, 2021) were sufficient to indicate that separate triathlon-specific tables of normative data for the BRUMS should be generated. Secondly, to more completely understand how the mood of triathletes impacts upon their performance, it would be necessary to assess mood at multiple intervals during the event and to link such “mood points” to the separate elements of performance, although the practical difficulties of doing this are considerable. Use of innovative technology will likely play a key role as a research tool via the development of automated mood tracking software incorporated into smart watches or other wearable devices, although at present such technology does not appear to be widely available. Thirdly, to use moods as an indicator of risk of mental health issues and thereby promote sustainable psychological well-being, investigation of the efficacy of regular mood profiling is encouraged. Online mood profiling systems, such as *In The Mood*^2^ (Terry et al., 2021a), provide open access to the BRUMS and include a personalized report of mood status along with evidence-based mood regulation strategies to help adjust undesirable moods. The effectiveness of such systems in helping to maintain psychological well-being is yet to be established empirically.

In conclusion, our study of age-group triathletes showed that their pre-event moods differed significantly from population norms and other athlete samples. Generally, triathletes reported more positive moods than other groups. Some gender and age band differences in mood were evident, with males and older triathletes reporting lower Tension than females and younger triathletes. Pre-event mood did not predict performance in triathlon, probably due to the considerable potential for substantial in-event mood fluctuation. Six distinct mood clusters were evident, almost identical to those identified previously in the literature. The low prevalence of the most negative profiles among triathletes indicated an association between participation in the sport and positive mental health.

## Data availability statement

The raw data supporting the conclusions of this article will be made available by the authors, without undue reservation.

## Ethics statement

The studies involving human participants were reviewed and approved by University of Southern Queensland’s Human Research Ethics Committee. The patients/participants provided their written informed consent to participate in this study.

## Author contributions

RP-S conception and design of study, acquisition of data, performed analysis, interpreted data, edited the manuscript, and acted as corresponding author. Final approval of the version to be published. Agreement to be accountable for all aspects of the work in ensuring that questions related to the accuracy or integrity of any part of the work are appropriately investigated and resolved. SB conception and design of study, acquisition of data, collated data, interpreted data, edited the manuscript. Final approval of the version to be published. Agreement to be accountable for all aspects of the work in ensuring that questions related to the accuracy or integrity of any part of the work are appropriately investigated and resolved. GL conception and design of study, acquisition of data, interpreted data, revised the manuscript critically for important intellectual content, final approval of the version to be published. Agreement to be accountable for all aspects of the work in ensuring that questions related to the accuracy or integrity of any part of the work are appropriately investigated and resolved. VV conducted much of the pre-cursor research upon which the study was based, provided critical triathlon-specific knowledge and interpretation of data, revised the manuscript critically for important intellectual content, final approval of the version to be published. Agreement to be accountable for all aspects of the work in ensuring that questions related to the accuracy or integrity of any part of the work are appropriately investigated and resolved. PT conception and design of study, supervised development of work, performed analysis, interpreted data, drafted the manuscript, and revised the manuscript critically for important intellectual content, final approval of the version to be published. Agreement to be accountable for all aspects of the work in ensuring that questions related to the accuracy or integrity of any part of the work are appropriately investigated and resolved. All authors contributed to the article and approved the submitted version.

## Funding

VV acknowledges the support of the Fundação para a Ciência e Tecnologia—as expressed by Grant UIDB/00447/2020 to CIPER—Centro Interdisciplinar para o Estudo da Performance Humana (unit 447).

## Conflict of interest

The authors declare that the research was conducted in the absence of any commercial or financial relationships that could be construed as a potential conflict of interest.

## Publisher’s note

All claims expressed in this article are solely those of the authors and do not necessarily represent those of their affiliated organizations, or those of the publisher, the editors and the reviewers. Any product that may be evaluated in this article, or claim that may be made by its manufacturer, is not guaranteed or endorsed by the publisher.

## Footnotes

^1^Vleck, V. E., and Panayides, M. (2018). Development of an algorithm to predict overuse injury in triathletes. Unpublished manuscript.

^2^ www.moodprofiling.com
